# A Genus Definition for *Bacteria* and *Archaea* Based on a Standard Genome Relatedness Index

**DOI:** 10.1128/mBio.02475-19

**Published:** 2020-01-14

**Authors:** R. A. Barco, G. M. Garrity, J. J. Scott, J. P. Amend, K. H. Nealson, D. Emerson

**Affiliations:** a Department of Earth Sciences, University of Southern California, Los Angeles, California, USA; b Department of Biological Sciences, University of Southern California, Los Angeles, California, USA; c Department of Microbiology and Molecular Genetics, Michigan State University, East Lansing, Michigan, USA; d Smithsonian Tropical Research Institute, Panama, Republic of Panama; e Bigelow Laboratory for Ocean Sciences, East Boothbay, Maine, USA; Oregon State University

**Keywords:** ANI, *Bacillus*, *Clostridium*, *Lactobacillus*, *Photorhabdus*, *Pseudomonas*, *Xenorhabdus*, delineation, demarcation, genus, systematics, taxonomy

## Abstract

Genus assignment is fundamental in the characterization of microbes, yet there is currently no unambiguous way to demarcate genera solely using standard genomic relatedness indices. Here, we propose an approach to demarcate genera that relies on the combined use of the average nucleotide identity, genome alignment fraction, and the distinction between type- and non-type species. More than 3,500 genomes representing type strains of species from >850 genera of either bacterial or archaeal lineages were tested. Over 140 genera were analyzed in detail within the taxonomic context of order/family. Significant genomic differences between members of a genus and type species of other genera in the same order/family were conserved in 94% of the cases. Nearly 90% (92% if polyphyletic genera are excluded) of the type strains were classified in agreement with current taxonomy. The 448 type strains that need reclassification directly impact 33% of the genera analyzed in detail. The results provide a first line of evidence that the combination of genomic indices provides added resolution to effectively demarcate genera within the taxonomic framework that is currently based on the 16S rRNA gene. We also identify the emergence of natural breakpoints at the genome level that can further help in the circumscription of taxa, increasing the proportion of directly impacted genera to at least 43% and pointing at inaccuracies on the use of the 16S rRNA gene as a taxonomic marker, despite its precision. Altogether, these results suggest that genomic coherence is an emergent property of genera in *Bacteria* and *Archaea*.

## Abstract (precis)

In recent decades, the taxonomy of *Bacteria* and *Archaea*, and therefore genus designation, has been largely based on the use of a single ribosomal gene, the 16S rRNA gene, as a taxonomic marker. We propose an approach to delineate genera that excludes the direct use of the 16S rRNA gene and focuses on a standard genome relatedness index, the average nucleotide identity. Our findings are of importance to the microbiology community because the emergent properties of *Bacteria* and *Archaea* that are identified in this study will help assign genera with higher taxonomic resolution.

## INTRODUCTION

At the time of writing, 20,768 bacterial and archaeal species/subspecies and over 3,500 genera with validly published names have been described in the taxonomic literature (1); however, based on a 16S rRNA guided-phylogenetic approach, there are over 200,000 bacterial and archaeal species and 60,000 genera so far detected in the SILVA database (2, 3). Sequence data stored in the Joint Genome Institute (JGI) database, which includes data from other databases, have increased exponentially over the past decade (4), with >76,000 genomes of bacterial and archaeal isolates, >9,000 metagenome-assembled genomes, and >4,000 single-cell amplified genomes being currently available (database accessed on 10 November 2019). There are >210,000 genome assemblies in GenBank (database accessed on 10 November 2019). Despite the increasing number of genomes, there were only <2,000 genomes of type strains that were publicly available at the start of this study, with 1,003 of these genomes only recently published (5). Currently, there are <9,000 nonredundant genomes of type strains that are publicly available (1). The number of genomes of type strains is presently increasing by over 1,000 per year, with recent plans to increase this number substantially (6); therefore, it is becoming easier to access data that represent taxa of interest. Consequently, this added layer of available information could aid in the formal characterization of microorganisms, of which an essential aspect is the proper assignment of genus and species (rule 12a of the International Code of Nomenclature of Prokaryotes [ICNP] [7]).

Historically, DNA-DNA hybridization (DDH) has been the “gold standard” for species delineation, with a DDH value of ≥70% being recognized as the species boundary between two strains (see reference 8 and other references therein). Stackebrandt and Goebel (9) conducted a correlation analysis between DDH values and 16S rRNA gene sequence identities, and based on this, they proposed a boundary of 16S rRNA gene sequence similarity of 97% for species delineation. This value, which is still largely used today for operational taxonomic unit (OTU)-based analysis of microbial communities, has been updated by Stackebrandt and Ebers (10) to a value between 98.7 and 99.0%, based on a greater amount of available sequence data. Subsequently, as genome sequencing has become common, whole-genome comparisons became possible, leading to the advent of genome relatedness indices such as average nucleotide identity (ANI) (11), amino acid identity (AAI) (12), and digital DDH (13). More recently, Kim et al. (14) proposed a 16S rRNA gene sequence similarity threshold value of 98.65% for species delineation, equating to ANI values of 95 to 96%, which in turn have been equated to the classical standard species delineation threshold DDH value of 70% (15, 16). A method that only relies on protein-coding genes (i.e., neither rRNA nor tRNA genes are included in analysis) is the Microbial Species Identifier (MiSI), which employs both alignment fractions (AF) and ANI for demarcation of species, recommending threshold AF and ANI values of 0.6 and 96.5% using complete or nearly complete genomes (17).

Despite these advancements in resolving species delineation, practical guidelines that incorporate genomic properties to demarcate genera have been lacking even though genus assignment is key to performing meaningful comparisons regarding the physiology, metabolism, and genomic potential of microbes. Methods to demarcate genera have been proposed that are based on either AAI (18) or the percentage of conserved proteins (POCP; 19). The former method provided a range of AAI values (65 to 72%) that were originally obtained by correlation to a now-outdated 16S rRNA gene identity threshold for genus. The POCP method directly relies on the 16S rRNA gene sequence, which is in some cases insensitive to evolutionary changes in the rest of the genome of a given organism, as revealed by different species sharing >99% identity over the length of this gene. This method also arbitrarily sets a genus boundary at a POCP value of 50%. Additionally, the generally used arbitrary genus threshold of 95% 16S rRNA gene identity has been recently revisited to a lower minimum value of 94.5%, with a median sequence identity of 96.4% and confidence interval of 94.55 to 95.05% (3). In borderline cases, interpretation of results may be unclear if there are no alternative ways to confirm genus assignment. This is also the case for microorganisms with multiple highly divergent 16S rRNA genes (20, 21). More recently, a taxonomy for *Bacteria* based on highly conserved protein-concatenated phylogeny and the normalization of ranks has been proposed (22). This normalized taxonomy uses ∼4% of a genome and directly relies on values of relative evolutionary divergence, a nonstandard index. Here, we propose a novel approach that builds on the MiSI method (17) and provides an objective, mathematically sound, and reproducible method of delimiting genera using whole-genome sequences and ANI, a standard genome relatedness index. In addition to identifying genus boundaries, we introduce the concept of the genus inflection point. We implement this approach by testing a variety of taxonomic groups of *Bacteria* and *Archaea*. Furthermore, evidence is presented to support the extensive rearrangement of Bacillus, Clostridium, Lactobacillus, and Photorhabdus, among other taxa, using a standard genomic index.

(An earlier version of this article was submitted to an online preprint archive [23].)

## RESULTS AND DISCUSSION

### General assessment.

A total of 3,525 genomes of nonredundant species representing 858 genera in 13 different phyla were used (see Data Set S1 in the supplemental material); 3,331 of these genomes were of type strains, with the rest belonging to species that are >99% identical in 16S rRNA gene sequence to a type strain. Genus demarcation boundaries were determined for each of the 144 genera that were analyzed in detail, at the taxonomic resolution of order/family (Data Set S2A). A subtotal of 3,077 of the species (>87%) were delineated in agreement with current taxonomy, following AF and ANI analyses, specifically the genus demarcation boundary. This value increases to 92% if known polyphyletic genera (e.g., *Bacillus*, *Clostridium*, *Lactobacillus*, and Pseudomonas) are excluded. These polyphyletic genera had the greatest number of non-type species at or below the genus demarcation boundary. In terms of disagreement with current taxonomy, Desulfovibrio had the highest proportion of non-type species at or below the genus demarcation boundary, indicating substantial taxonomic issues with this genus as well.

10.1128/mBio.02475-19.2 DATA SET S1 List of genomes used in this study. Taxa in bold indicate the taxonomic resolution at which the group was analyzed. Download Data Set S1, XLSX file, 0.5 MB. Copyright © 2020 Barco et al. 2020 Barco et al. This content is distributed under the terms of the Creative Commons Attribution 4.0 International license.

10.1128/mBio.02475-19.3 DATA SET S2 (a) Summary of taxonomic groups and genomes included in this study. Taxa in bold indicate the taxonomic resolution at which the group was analyzed. For a detailed species list including accession numbers, see Data Set S1. (b) AF and ANI mean and median values associated with genus demarcation boundaries in *Archaea* and *Bacteria*. (c) List of estimated genus inflection points. For accuracy, this list only includes the genus inflection points that were consistent with Gompertz and logistic regressions and had, at a minimum, *R*^2^ values approximating 0.90 (quartic regression). Download Data Set S2, XLSX file, 0.1 MB. Copyright © 2020 Barco et al. 2020 Barco et al. This content is distributed under the terms of the Creative Commons Attribution 4.0 International license.

**FIG 1:**
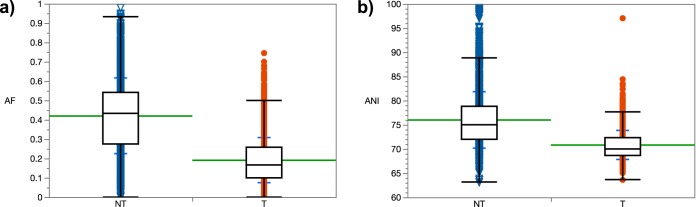
Distribution of AF (a) and ANI (b) values of type (T; *n* = 2,382) and non-type (NT; *n* = 2,571) species compared to a primary reference in their respective order/family in *Bacteria*. Significant differences were seen between T and NT species in both cases (nonparametric Wilcoxon test, *P* < 0.0001). The green line indicates the mean. The blue lines show the standard deviation from the mean.

**FIG 2:**
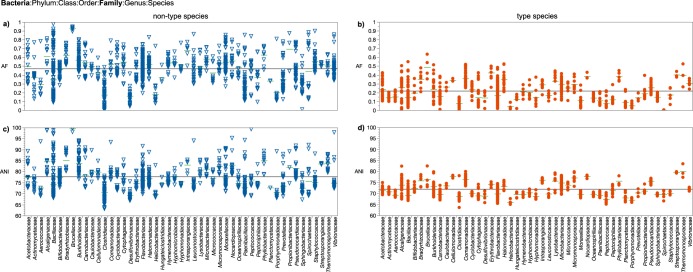
AF (a and b) and ANI (c and d) values of type (*n* = 1,641) and non-type (*n* = 1,554) species compared to primary references within specific taxonomic families in *Bacteria*. The mean AF or ANI value is shown by a green line in a–d. The mean of means is denoted by the black line in a–d.

The AF and ANI means of the type and non-type species clusters were significantly different (*P* < 0.0001) at the taxonomic level of domain for bacterial (Fig. 1) and archaeal (see Fig. S1) genera. For the majority of the genera (94%), the combination of AF and ANI genomic indices resulted in clustering of type and non-type species. The AF and ANI means of these clusters were significantly different (*P* < 0.05) in 94% and 90% of the cases for AF and ANI, respectively (Data Set S2A). Figure 2 shows the distribution of AF and ANI values at the taxonomic level of family in *Bacteria* (see Fig. S2 and S3 for distributions at the taxonomic level of order in *Bacteria* and of order and family in *Archaea*, respectively). The 448 identified type strains that would need reclassification directly impact 48 of the 144 genera analyzed in this study. The number of genera that are impacted increases to 62 when the estimated genus inflection point (discussed in detail below) is used.

10.1128/mBio.02475-19.5 FIG S1 Distribution of AF and ANI values of type (T; *n* = 82) and non-type (NT; *n* = 96) species compared to a primary reference in their respective order/family in *Archaea*. The green line indicates the mean. The blue lines show the standard deviation from the mean. Download FIG S1, EPS file, 0.9 MB. Copyright © 2020 Barco et al. 2020 Barco et al. This content is distributed under the terms of the Creative Commons Attribution 4.0 International license.

10.1128/mBio.02475-19.6 FIG S2 (a to d) AF (a and b) and ANI (c and d) values of type (T; *n* = 2,382) and non-type (NT; *n* = 2,571) species compared to primary references within specific taxonomic orders in *Bacteria*. Download FIG S2, EPS file, 0.7 MB. Copyright © 2020 Barco et al. 2020 Barco et al. This content is distributed under the terms of the Creative Commons Attribution 4.0 International license.

The means and medians of all the AF and ANI values associated with genus demarcation boundaries as they pertain to *Bacteria* and *Archaea* are included in Data Set S2B. Altogether (*n* = 144), the AF values of genus demarcation boundaries have a mean of 0.331 (95% confidence interval [CI], 0.308 to 0.354), with a median of 0.345 (25% quartile, 0.206; 75% quartile, 0.444). The ANI values of genus demarcation boundaries have a mean of 73.98% (95% CI, 73.34% to 74.62%), with a median of 73.11% (25% quartile, 70.85%; 75% quartile, 76.56%).

The AF values of the estimated genus inflection points obtained in this study (*n* = 28; all from genera in *Bacteria*; Data Set S2C) have a mean of 0.333 (95% CI, 0.305 to 0.362), with a median of 0.349 (25% quartile, 0.281; 75% quartile, 0.371). These values are nearly identical to the AF mean (0.330) and median (0.345) of the genus demarcation boundaries. The ANI values of the estimated genus inflection points obtained in this study have a mean of 73.10% (95% CI, 72.50% to 73.70%), with a median of 73.08% (25% quartile, 72.57%; 75% quartile, 73.93%). These values are also in close proximity to the mean (73.98%) and median (73.11%) ANI values of the genus demarcation boundaries.

We encourage the use of the genus demarcation boundary in conjunction with the estimated genus inflection point that is specific to the taxon that is being analyzed (Data Set S2A). The mean and median values that were obtained are only presented here for comparison purposes and in the case that a genus demarcation boundary or estimated genus inflection point cannot be determined (e.g., due to lack of type species and/or type strains). Several representative cases will be discussed in more detail below.

### *Bacteria*.

**FIG 3:**
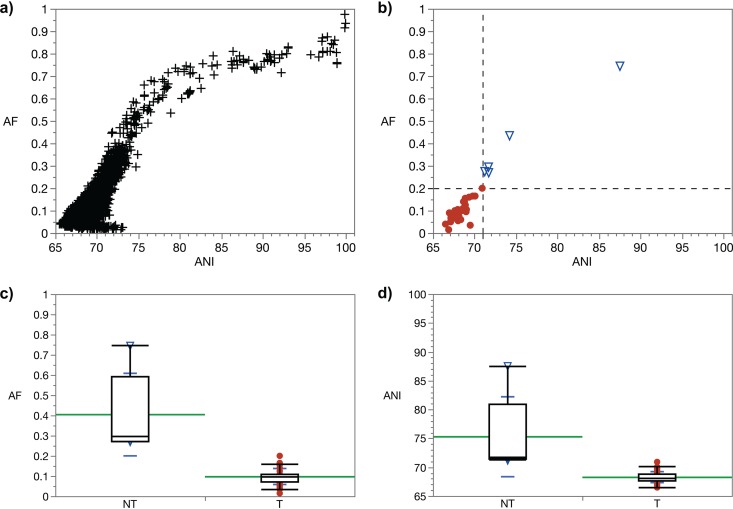
AF and ANI pairwise genome comparisons in the order *Lactobacillales*. (a) Type and non-type strains/species of 15 different genera in the order *Lactobacillales* were pairwise compared (*n* = 6,090 comparisons). (b) Type species (*n* = 37) of genera within the order *Lactobacillales* (circles) and non-type species (*n* = 6) of the genus *Lactococcus* (in order *Lactobacillales*; triangles) were pairwise compared only to the primary reference, type species Lactococcus lactis subsp. *lactis* ATCC 19435^T^. The bottom-left quadrant demarcates the boundary between type and non-type species. (c and d) Boxplot diagrams of AF (c) and ANI (d) values as related to non-type (NT) species of the genus *Lactococcus* and type (T) species of genera within the order *Lactobacillales* (nonparametric Wilcoxon test, *P* = 0.0003 in both cases). Means are shown in green. Standard deviations are shown in blue.

To initially test our approach with Gram-positive bacteria, the order *Lactobacillales* was chosen since it contains a number of well-characterized genera and species of economic importance in human and animal health. A continuum of AF and ANI values characterizes a typical result when intergeneric and intrageneric species of the order *Lactobacillales* (e.g., all against all) are compared without distinguishing between type and non-type strains or species (Fig. 3a). Because there is no primary taxonomic reference genome to compare to, differentiation between different taxonomic groups is not possible. However, when the type species of *Lactococcus*, L. lactis subsp. lactis ATCC 19435^T^ (68), is used as the primary reference, a clear distinction can be made between type species and non-type species (Fig. 3b). The type species of genera in *Lactobacillales* form a distinct cluster toward lower AF and ANI values, while the non-type species of *Lactococcus* cluster toward higher AF and ANI values (Fig. 3c and d). There is no overlap between these two clusters. All species were correctly classified into their corresponding groups by using the genus demarcation boundary.

**FIG 4:**
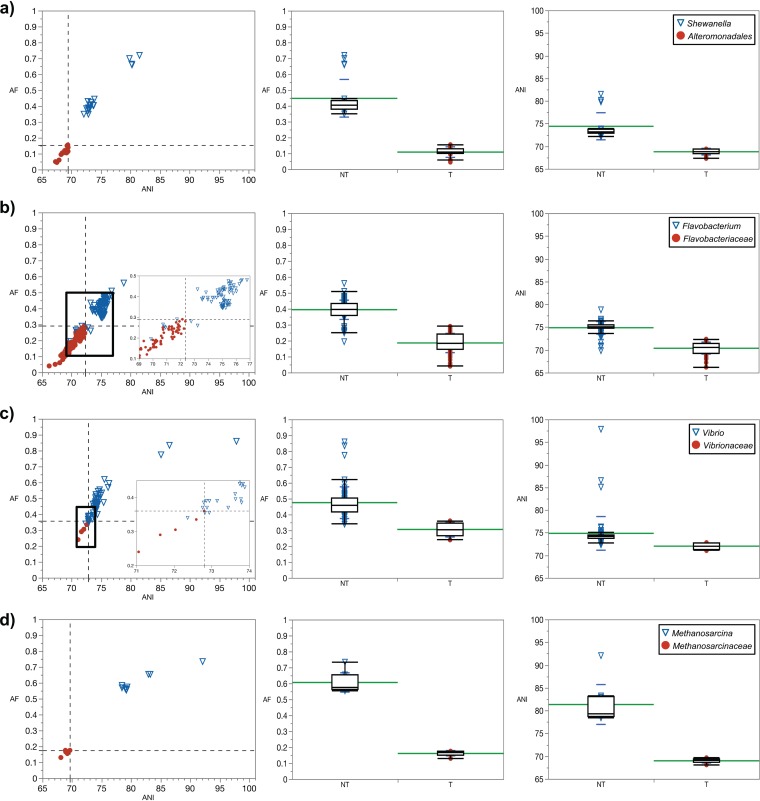
(a to d) Pairwise genome comparisons to primary references. (a) Type species of the genus *Shewanella*, S. putrefaciens JCM 20190^T^. Circles, type species (*n* = 19) of genera within the order *Alteromonadales*; triangles, non-type species (*n* = 23) of the genus *Shewanella* (nonparametric Wilcoxon test, *P* < 0.0001 with AF or ANI). (b) Type species of the genus *Flavobacterium*, *F. aquatile* LMG 4008^T^. Circles, type species (*n* = 69) of genera within the family *Flavobacteriaceae*; triangles, non-type species (*n* = 83) of the genus *Flavobacterium* (*P* < 0.0001 with AF or ANI). The inset shows a zoomed-in boxed area. (c) Type species of the genus *Vibrio*, V. cholerae ATCC 14035^T^. Circles, type species (*n* = 5) of genera within the family *Vibrionaceae*; triangles: non-type *Vibrio* spp. (*n* = 65; *P* = 0.0003 with AF or ANI). The inset shows zoomed-in boxed area. (d) Type species of the genus *Methanosarcina*, *M. barkeri* JCM 10043^T^. Circles, type species (*n* = 6) of genera within the family *Methanosarcinaceae*; triangles, non-type *Methanosarcina* spp. (*n* = 9; *P* = 0.0017 with AF; *P* = 0.0018 with ANI). In all cases, the bottom-left quadrant demarcates the boundary between type and non-type species. Shown are boxplots of AF (center) and ANI (right) values as related to non-type (NT) species of genus analyzed and type species (T) of genera within the order or family analyzed. Means are shown in green. Standard deviations are shown in blue.

To test our approach with Gram-negative microorganisms, type species of genera in the order *Alteromonadales* and non-type Shewanella spp. were pairwise compared to the type species Shewanella putrefaciens JCM 20190^T^, which is the primary reference (Fig. 4a). A distinct clustering is seen between the type species and non-type species. All species were correctly classified into their corresponding groups by using the genus demarcation boundary.

Additional examples show that this trend is also seen with taxonomic families (Fig. 2 and 4b). The *Flavobacteriaceae* were investigated as an example of a diverse family with one of the largest number of validly published genera (currently >160). Despite this, the separate clustering of type species and non-type species was conserved. When the type species of the genus Flavobacterium, Flavobacterium aquatile LMG 4008^T^, is used as the primary reference, all type species (i.e., with available genomes) in the *Flavobacteriaceae* clustered toward lower AF and ANI values, while the vast majority of the non-type species of *Flavobacterium* clustered toward higher AF and ANI values. The five *Flavobacterium* spp. that were positioned below the AF and ANI genus demarcation boundary had 16S rRNA gene sequence identities of <94% to the primary reference. In a separate example, the type species Vibrio cholerae ATCC 14035^T^ was used as the primary reference and compared to non-type species in the genus *Vibrio* and other type species of genera in the family *Vibrionaceae* (Fig. 4c). Clustering is seen between the type species and non-type species. All species were correctly classified into their corresponding groups by using the genus demarcation boundary, with the exception of Vibrio caribbeanicus ATCC BAA-2122^T^, which has a 16S rRNA gene identity of 92.9% to V. cholerae ATCC 14035^T^.

### *Archaea*.

This approach was also tested on an archaeal family, *Methanosarcinaceae*, in the phylum *Euryarchaeota* (Fig. 4d). The primary reference was the type species Methanosarcina barkeri JCM 10043^T^. The results were consistent with previous examples of bacterial taxa displaying clustering of non-type species within a genus separately from type species of other genera within the same family. As in previous examples, all species were classified into their correct higher taxa by using the genus demarcation boundary. Similar results were obtained in the family *Haloferacaceae*. Additionally, the family *Thermoproteaceae* in the phylum *Crenarchaeota* was tested, with Pyrobaculum islandicum DSM 4184^T^ as a primary reference. A significant distinction between type and non-type species was seen with AF (*P* = 0.0369) but not with ANI (*P* = 0.3682) (Data Set S2A). Despite this, there was no overlap of the type/non-type clusters when both AF and ANI were used in combination, and all of the species were classified in agreement with current taxonomy.

### Selected case studies.

In the remainder of the manuscript, we will focus on five selected case studies, each of which has longstanding, historical, taxonomic issues. These cases will be discussed in more detail, with the goal of guiding the reader toward an interpretation of similar scenarios in other taxa. These case studies, along with the examples described above, provide a variety of different scenarios that can be analyzed using our approach.

**FIG 5:**
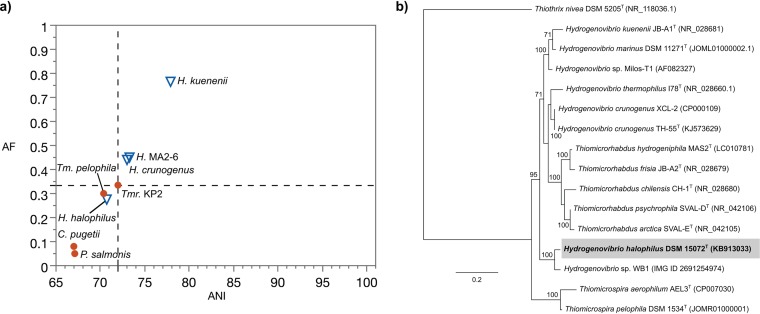
Analysis of Hydrogenovibrio, as rearranged by Boden et al. (26). (a) Pairwise genome comparisons to the primary reference Hydrogenovibrio marinus DSM 11271^T^. Non-type species within the genus Hydrogenovibrio are shown in triangles. The type strain *H. thermophilus* I78^T^ does not have a sequenced genome; therefore, Hydrogenovibrio sp. strain MA2-6 (>99% pairwise identity in 16S rRNA gene sequence) was used instead. Similarly, *H. crunogenus* XCL-2 (>99% pairwise identity in 16S rRNA gene sequence) (69) is used instead of the type strain *H. crunogenus* TH-55^T^, as TH-55^T^ does not have a sequenced genome. The type species of genera within the family *Piscirickettsiaceae* are shown in circles. The bottom-left quadrant demarcates the boundary between type and non-type species. (b) Maximum likelihood phylogenetic tree based on an alignment of 16S rRNA genes indicating the phylogenetic positioning of *H. halophilus* DSM 15072^T^. The scale bar indicates 20% sequence divergence. Bootstrap values >70% are shown at the nodes. Accession numbers are shown to the right in parentheses. Tm, *Thiomicrospira*; Tmr, *Thiomicrorhabdus.*

Genus assignment in the recently rearranged Hydrogenovibrio-Thioalkalimicrobium-Thiomicrospira cluster was tested as an example of a bacterial group with historical taxonomic issues (24, 25) (Fig. S4A). Boden et al. (26) recently provided a detailed evaluation of the characteristics of this cluster, which falls within the radiation of the *Piscirickettsiaceae* and is proposed to place many of the species into different genera. We tested these newly proposed assignments using the approach described above and the MiSI method. When testing genus assignment to Hydrogenovibrio, the type species Hydrogenovibrio marinus DSM 11271^T^ was used as a primary reference (Fig. 5a). The single data point that crossed the genus demarcation boundary belonged to Hydrogenovibrio halophilus DSM 15072^T^. Aside from this data point, there was no overlap of AF and ANI values between the type- and non-type species, indicating support for the assignment of H. crunogenus, H. thermophilus, and H. kuenenii to the genus Hydrogenovibrio. Similarly, the proposed new genus Thiomicrorhabdus (26) was also tested (Fig. S4B). The results indicate that the new classification is supported by AF and ANI values, as the type species of genera in the family *Piscirickettsiaceae* and *Thiomicrorhabdus* spp. remain well separated. The reclassification of *Thioalkalimicrobium* spp. to *Thiomicrospira* spp. (26) is also supported by ANI and AF values (Fig. S4C), as these species form a cluster that is well separated from the type species of genera in the family *Piscirickettsiaceae*.

10.1128/mBio.02475-19.7 FIG S3 (a to h) Distribution of AF and ANI values in *Archaea* at the order (a to d) and family (e to h) levels. (a to d) AF (a and b) and ANI (c and d) values of non-type species (*n* = 96) and type species (*n* = 82) compared to primary references within specific taxonomic orders in *Archaea*. (e to h) AF (e and f) and ANI (g and h) values of type (T; *n* = 69) and non-type (NT; *n*= 73) species compared to primary references within specific taxonomic families in *Archaea*. Download FIG S3, EPS file, 2.6 MB. Copyright © 2020 Barco et al. 2020 Barco et al. This content is distributed under the terms of the Creative Commons Attribution 4.0 International license.

10.1128/mBio.02475-19.8 FIG S4 Pairwise genome comparisons of *Thiomicrospira* and *Thiomicrorhabdus*. (a) Pairwise genome comparisons to the type species Thiomicrospira pelophila DSM 1534^T^ using the old classification scheme (i.e., prior to rearrangement by Boden et al. [26]) show clear taxonomic issues and no distinct clustering between type and non-type species. Non-type species within the genus *Thiomicrospira* are shown in triangles. Type species of genera within the family *Piscirickettsiaceae* are shown in circles. Inset shows a zoomed-in boxed area. (b) Pairwise genome comparisons to *Thiomicrorhabdus* sp. strain KP2 (>99% pairwise identity to the 16S rRNA gene sequence of the type species Thiomicrorhabdus frisia JB-A2^T^, which does not have a sequenced genome) using the new reclassification scheme by Boden et al. (26). Non-type species within the genus *Thiomicrorhabdus* are shown in triangles. Type species of genera within the family *Piscirickettsiaceae* are shown in circles. The bottom-left quadrant demarcates the boundary between type and non-type species. (c) Pairwise genome comparisons to Thiomicrospira pelophila DSM 1534^T^ using the new reclassification scheme by Boden et al. (26). Non-type species within the genus *Thiomicrospira* are shown in triangles. Type species of genera within the family *Piscirickettsiaceae* are shown in circles, with the exception of Thiomicrorhabdus frisia JB-A2^T^, which does not have a sequenced genome; *Thiomicrorhabdus* sp. KP2 (>99% pairwise identity to the 16S rRNA gene sequence) was used instead. The bottom-left quadrant demarcates the boundaries between type and non-type species. Download FIG S4, EPS file, 0.3 MB. Copyright © 2020 Barco et al. 2020 Barco et al. This content is distributed under the terms of the Creative Commons Attribution 4.0 International license.

Our approach has identified a potential misclassification of *H. halophilus* (basonym Thiomicrospira halophila) because it clusters with the type species of genera in the family *Piscirickettsiaceae* other than Hydrogenovibrio. Below, we show additional evidence that could support a potential reclassification of *H. halophilus*. A closer look at the multiple 16S rRNA gene sequences of *H. halophilus* revealed that they have 94.0 to 94.2% pairwise identities to the type species, *H. marinus* DSM 11271^T^. Our results differ from those of Boden et al., who reported 95.7% sequence identity (26) between *H. halophilus* and *H. marinus*. The difference in identity appears to be due to differences in the 16S rRNA gene sequence between the near-full-length version of Sorokin et al. (1,420 bp, GenBank accession number DQ390450 [27] and the full-length versions of this gene (1,439 bp each; IMG identifiers [IDs] 2518265101 and 2518265550) originating from a draft genome (28) and used in this study. The phylogenetic placement of this organism also supports the idea that *H. halophilus* is distinctly positioned in its own clade as it branches away from the cluster of other Hydrogenovibrio spp. (Fig. 5b). One differentiating characteristic of *H. halophilus* is the DNA G+C content of 56.6%, much higher than the ca. 44% of the other newly classified Hydrogenovibrio spp. Another major differentiating aspect is the higher NaCl optimum/maximum of 1.5 M/3.5 M for *H. halophilus* versus the lower 0.2 to 0.5/0.6 to 1.2 M for other Hydrogenovibrio spp. Therefore, based on AF and ANI results and molecular, phylogenetic, and physiological evidence, the assignment of *H. halophilus* to a new genus is justifiable. Beyond the taxonomic issue with *H. halophilus*, we also note the relatively low AF and ANI values of *H. crunogenus* and Hydrogenovibrio sp. strain MA2-6 (a strain of *H. thermophilus*) in relation to *H. marinus*. These results in conjunction with the low bootstrap values for branches associated with *H. crunogenus* and *H. thermophilus* (<70% in reference 26 and in this study) suggest that there are other taxonomic issues that cannot be resolved at this time in this genus, in part due to the lack of genomes of type strains (see below for discussion on the genus inflection point).

**FIG 6:**
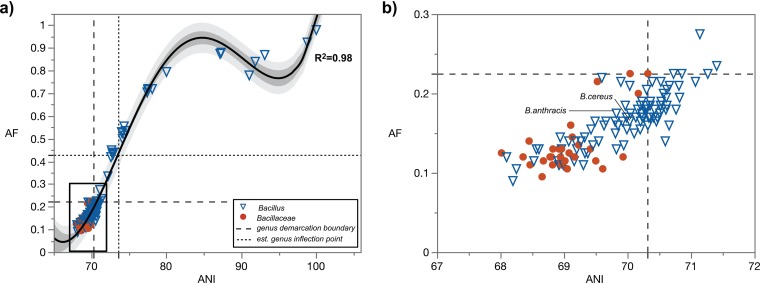
Pairwise genome comparisons to the type species of the genus *Bacillus*, B. subtilis ATCC 6051^T^. Circles, type species (*n* = 30) of genera within the family *Bacillaceae*; triangles, non-type *Bacillus* spp. (*n* = 117). (a) The regression line is based on quartic function, with the data set including the AF and ANI value of 1:100. The dark-gray-shaded area indicates the 95% confidence interval of the trendline. The light-gray-shaded area indicates the 95% confidence interval of AF and ANI values. The long dash line demarcates the genus boundary (left quadrant). The short dash line (left quadrant) extends from the estimated genus inflection point. (b) Zoomed-in boxed area shown in panel a; est., estimated.

The *Bacillaceae* were also investigated as an example of a family that is medically and commercially relevant (Fig. 6) but problematic in terms of taxonomy. Generally, there were significant differences between non-type *Bacillus* species and type species of genera in the family *Bacillaceae* when B. subtilis 6051^T^ (29) was used as a primary reference (Data Set S2A). However, approximately half (59 out of 117) of the non-type *Bacillus* spp. clustered with type species, which strongly suggests that the genus *Bacillus* is in need of taxonomic revision. Of note, the human pathogens Bacillus anthracis and Bacillus cereus cluster with type species of genera in *Bacillaceae*, indicating that they are at least as genomically different from B. subtilis as are other type species in this family, warranting a taxonomic rearrangement. As shown above with *H. halophilus*, a similar argument could be made for the taxonomic rearrangement of each one of these species (e.g., <94.5% identity in 16S rRNA gene sequence to the type species B. subtilis 6051). Compared to our analyses of other taxonomic groups, these results are atypical in the sense that the non-type species cluster considerably overlaps the type species cluster. We highlight the fact that a large number of these specimens were designated non-type species of *Bacillus* prior to the advent of the universal use of 16S rRNA gene as a taxonomic marker in the 1990s (i.e., >20% of all type strains of non-type *Bacillus* spp. with available genomes in our data set). For example, B. anthracis was first described in 1872 (30) and appears on the Approved Lists of Bacterial Names (31). When the primary reference is changed to Anoxybacillus pushchinoensis K1^T^, a type species of a genus established in 2000 (32), using the taxonomic framework of the 16S rRNA gene, the clustering of type species of genera in *Bacillaceae* and non-type species of *Anoxybacillus* is almost without overlap, with the genus demarcation boundary delineating 97% of the species in agreement with current taxonomy (Data Set S2A). Another example is Lysinibacillus, which was proposed and validly published in 2007 (33), showing distinct clustering between type species and non-type species in full agreement with current taxonomy.

The AF and ANI plots reveal different rates of change at the DNA level. In the few cases in which there are sufficient samples to analyze and/or the distribution of values is more widespread, the AF and ANI plots reveal a polynomial shape approximating a quartic function, with *R*^2^ values generally at >0.96. A second derivative of this function results in a quadratic function that can be used to detect an inflection point with precision. Estimates based on several examples in this study indicate two inflection points. The overall mean of the first inflection point (AF, 0.333; ANI, 73.10%; Data Set S2C) generally approximates the mean genus demarcation boundary of all cases herein presented (AF, 0.331; ANI, 73.98%; Data Set S2B). Estimations of this genus inflection point via logistic and Gompertz functions generally agree with the estimations by the preferred quartic function, and their use is recommended for accuracy. The second inflection point, at 89 to 92% ANI, is consistently lower than the current ANI species threshold (≥95% ANI) and is seen with the quartic function in each of the cases in which the genus inflection point was able to be estimated. Related to this, genetic discontinuity between 80 and 95% ANI has been previously reported in various studies that included analysis of metagenomes and genomes of isolates (34–37). Recent results obtained by Delmont et al. (38) reported delineation of SAR 11 populations at ANI values lower than 95%, consistent with the ANI values of the second inflection points that were observed in this study. Because the second inflection point potentially deals with species delineation, detailed exploration (e.g., using subspecies type strains) and discussion of this topic are outside the scope of this study. However, it is noted that ANI values of 89 to 92% largely correspond to 16S rRNA gene identity values of ≥98.65% (see Fig. 3 in reference 14). Also, it is noted that there is a continuum of diversity at the genus level (e.g., as opposed to what is seen at the species level with genetic discontinuity) in addition to clustering (see Fig. 2), adding support to the idea proposed by Palmer et al. (39) that genetic continuum and genomic cohesiveness are not mutually exclusive, at least at the genus level.

**FIG 7:**
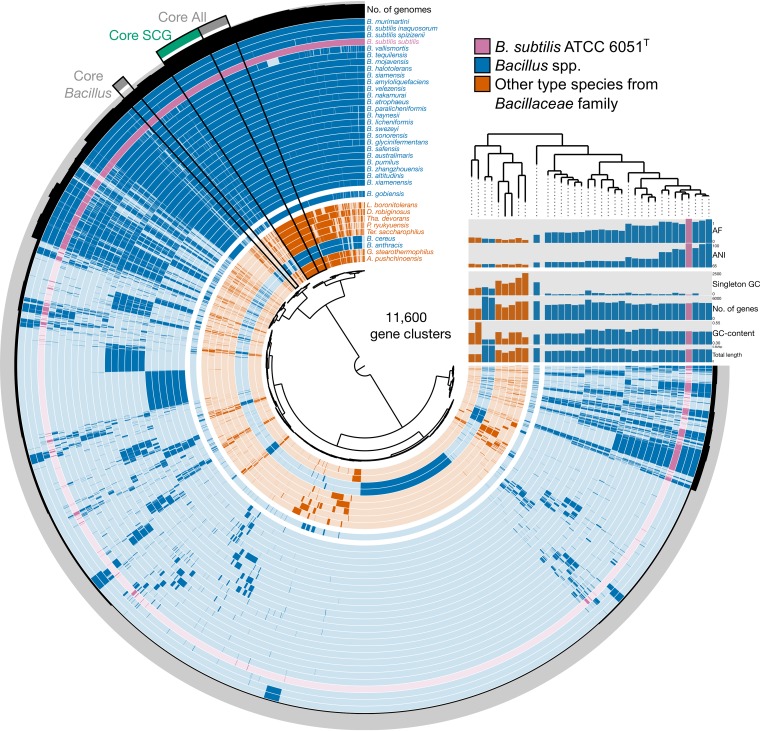
Pangenome of *Bacillus* (in blue). Type species of genera in the family *Bacillaceae* (i.e., other than B. subtilis ATCC 6051^T^) that are relatively close to the genus demarcation AF and ANI threshold (see Fig. 6) are shown in orange. The “Core *Bacillus*” bin includes all *Bacillus* spp. with AF and ANI values that are either above the estimated genus inflection point or within the 99% confidence interval of the estimate. “Core *Bacillus*” does not include *B. gobiensis* (i.e., AF and ANI values below estimated genus inflection point), B. anthracis, or B. cereus (i.e., B. anthracis and B. cereus have AF and ANI values below the genus demarcation boundary). ANI and AF values were obtained via pairwise genome comparisons to the primary reference, B. subtilis ATCC 6051^T^ (in purple). Hierarchical clustering was performed on the presence/absence of gene clusters using Euclidean distance and Ward linkage. SCG, single-copy genes. Singleton GC, singleton gene clusters.

The estimated genus inflection point serves as a practical guide for maximum AF and ANI values of a genus demarcation boundary and for the identification of a transition zone. Ideally, and assuming abundance of genomes of type strains and, consequently, data points, the estimated genus inflection point should closely match the genus demarcation boundary. As an example, the genus inflection point in *Bacillus* was estimated via the quartic function with AF of 0.430 and ANI of 73.57% (Fig. 6a). However, the majority of the non-type *Bacillus* spp. (99 out of 117 [85%]) fall below this inflection point at lower AF and ANI values, suggesting a potentially profound underestimation of genus diversity within the family *Bacillaceae*. An analysis of the pangenome of *Bacillus* using this estimated genus inflection point as a guide shows that only non-type *Bacillus* spp. above the genus inflection point cluster closely with the type species B. subtilis (the primary reference), while B. anthracis and B. cereus, which are below both the estimated genus inflection point and genus demarcation boundary, cluster with type species of other genera in *Bacillaceae* (Fig. 7), corroborating, as pointed out above, that these two pathogens warrant reclassification and renaming. However, doing so would require considerable care so as not to raise objections over safety issues and a push for conservation of the current names over newly proposed names (see rule 56a in reference 5 and opinion 60 in reference 7). These results were further corroborated by phylogenomic analysis of *Bacillaceae* (Fig. 7).

It is important to note that the genus inflection point is estimated based on nonlinear regression and only serves as a guide for future taxonomic designation. This is particularly important in the case of a new taxonomic designation with a genome displaying AF and ANI values that are right above the effective genus demarcation boundary; is it a novel type species of a new genus or a novel non-type species of an existing genus? Having an estimated genus inflection point helps in making this decision. Whereas the genus demarcation boundary is a hard boundary, the genus inflection point represents a soft boundary that highlights a region where the rate of change is starting to decrease at the genome level. Toward higher ANI values, this rate then starts to increase, which would correspond to a species inflection point. Thus far, the genus inflection point could be properly estimated only for a few taxonomic groups due to a lack of genomes of type strains and/or actual isolates. Bacillus pumilus, Bacillus safensis, Bacillus altitudinis, Bacillus zhangzhouensis, Bacillus xiamenensis, and Bacillus australimaris are borderline species positioned within the 99% confidence interval of the estimated genus inflection point. Their 16S rRNA gene identities in relation to the primary reference range from 96.8% to 97.4%, suggesting that the estimated genus inflection point of *Bacillus* corresponds to a value that is much higher than the current genus threshold based on 16S rRNA gene identity (94.5%).

The taxonomic placements of species within *Bacillus* were also compared to a recent taxonomy that is based on highly conserved, protein-concatenated phylogeny (Genome Taxonomy Database [GTDB] [22]). There are currently >280 *Bacillus* spp. in the literature with validly published names, but <50% of these species have available sequenced genomes of their type strains. The MiSI method and GTDB taxonomy (accessed on 7 May 2019) indicate that there are 19 and 26 species within *Bacillus*, respectively (Data Set S3A). The 19 species placed within *Bacillus* by the MiSI method represent the type strains that are above the genus inflection point. However, if borderline species (i.e., within the confidence interval of the estimated genus inflection point) are also included, the number of species within *Bacillus* increases to 26. The great majority of the GTDB classifications of species within *Bacillus* (i.e., “d_Bacteria; p_Firmicutes; c_Bacilli; o_Bacillales; f_Bacillaceae; g_Bacillus” in GTDB) were supported by the MiSI method if borderline species were taken into account, with two exceptions, as follows: (i) *B. gobiensis*, which is considerably below the estimated genus inflection point and outside the confidence interval, and (ii) “B. cellulasensis,” which is not a species with a validly published name.

10.1128/mBio.02475-19.4 DATA SET S3 Comparisons of species designations to GTDB taxonomy. (a) Species composition of *Bacillus* based on MiSI and GTDB (only considering type strains). (b) Species composition of *Clostridium* based on MiSI and GTDB (only considering type strains, unless noted otherwise). (c) Species composition of *Lactobacillus* based on MiSI and GTDB (only considering type strains with available genomes). Download Data Set S3, XLSX file, 0.1 MB. Copyright © 2020 Barco et al. 2020 Barco et al. This content is distributed under the terms of the Creative Commons Attribution 4.0 International license.

Clostridium is a genus with historical taxonomic issues. There are currently 165 *Clostridium* spp. but only 86 available, nonredundant, sequenced genomes of type strains. The MiSI method identified 36 of these *Clostridium* spp. to be at or below the genus demarcation boundary, indicating numerous taxonomic issues with this genus. In addition to this, there is a lack of available genomes of *Clostridium* spp. with ANI values between 80 and 100%. The genus inflection point of *Clostridium* is estimated at an AF of 0.252 and ANI of 72.72%. There are 21 *Clostridium* spp. at or above this inflection point. A comparison to GTDB taxonomy (i.e., limited to type strains; *n* = 19 in GTDB) again reveals a high level of congruency with the MiSI method, matching 74% (14/19; Data Set S3B) of the *Clostridium* designations. The points of disagreement are limited to Clostridium cavendishii, Clostridium fallax, Clostridium perfringens, Clostridium ventriculi (note that this is a validly published name but illegitimate [40]), and Clostridium intestinale, with C. intestinale being a borderline case (i.e., within a 99% confidence interval of the estimated genus inflection point). An updated taxonomy based on phylogenomics has recently proposed to include these five species in *Clostridium* cluster I (*sensu stricto*), in agreement with our results (41).

**FIG 8:**
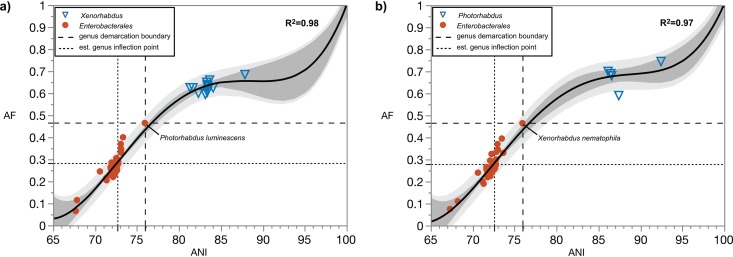
Close relationship between *Photorhabdus* and *Xenorhabdus* as revealed by genome relatedness indices. In both panels, the regression line is based on quartic function. The dark-gray-shaded area indicates the 95% confidence interval of the trendline. The light gray shaded area indicates the 95% confidence interval of AF and ANI values. The data set includes the AF and ANI value of 1:100. (a) Pairwise genome comparisons to the type species of the genus *Xenorhabdus*, *X. nematophila* ATCC 19061^T^. Circles, type species (*n* = 44) of genera within the order *Enterobacterales*; triangles, non-type *Xenorhabdus* spp. (*n* = 13). (b) Pairwise genome comparisons to the type species of the genus *Photorhabdus*, *P. luminescens* DSM 3368^T^. Circles, type species (*n* = 44) of genera within the order *Enterobacterales*; triangles, non-type *Photorhabdus* spp. (*n* = 5). The long dash line demarcates the genus boundary (left quadrant). The short dash line (left quadrant) extends from the estimated genus inflection point.

Xenorhabdus (42) has been the focus of multiple taxonomic evaluations. Xenorhabdus luminescens had been reclassified to the genus *Photorhabdus*, as P. luminescens (43), based in part on DNA-DNA hybridization analysis, even though this method is not necessarily suitable for genus demarcation. Separate phylogenetic analyses of both *Xenorhabdus* and *Photorhabdus* spp. indicated that in most cases, *P. luminescens* grouped within the *Xenorhabdus* cluster (44, 45). Now that the genome of *P. luminescens* has been sequenced, it is evident that it contains multiple 16S rRNA genes ranging in identity from 99 to 100%. Pairwise comparisons of these genes to the 16S rRNA genes of the type species Xenorhabdus nematophila result in identities ranging from 94 to 95%, with a borderline status based on the current 16S rRNA gene sequence identity genus threshold of 94.5%. If analysis of AF and ANI is done on *Xenorhabdus* using *X. nematophila* ATCC 19061^T^ (70) as the reference, *P. luminescens* sets the genus demarcation boundary (Fig. 8a), with AF and ANI values that are atypically high (AF, 0.465; ANI, 75.93%) for a type species of a genus other than the primary reference. Alternatively, if the same analysis is done on *Photorhabdus* using *P. luminescens* DSM 3368^T^ as the reference, *X. nematophila* sets the genus demarcation boundary with identical and atypical high AF and ANI values (Fig. 8b). Despite this, the AF and ANI analysis does not conflict with current classification of these taxa, as non-type species cluster above the genus boundary in either case, and type species of genera in *Enterobacterales* remain separated from this cluster. However, if the genus inflection point is estimated for *Xenorhabdus*, it is evident that *P. luminescens* is well above it, suggesting that *P. luminescens* should be in the same genus as the reference *X. nematophila* (Fig. 8a). If the inflection point is estimated for *Photorhabdus*, similar results are obtained, with *X. nematophila* clearly placed above the estimated inflection point (Fig. 8b). At a minimum, the combined results suggest that *X. nematophila* and *P. luminescens* belong to the same genus. Based on priority of publication, *P. luminescens* should be considered a later homotypic synonym of *X. luminescens* (42). It is noteworthy that bioluminescence is usually one of the properties that is highlighted as a differentiating characteristic of *Photorhabdus*; however, at least one strain in this genus, *P. luminescens* Q-614, is not bioluminescent (46). Pangenomic analysis of *Xenorhabdus* spp. further shows that none of the type species in *Enterobacterales* cluster with *Xenorhabdus*, except *P. luminescens*, suggesting that *Photorhabdus* is not as genomically different from *X. nematophila* as are other type species in *Enterobacterales*. This is seen despite the close relatedness of Arsenophonus, Morganella, Moellerella, Proteus, and Providencia to *Xenorhabdus*, which is evident in the phylogenomic analysis using either single-copy genes or ribosomal genes (Fig. S5).

10.1128/mBio.02475-19.9 FIG S5 Genome clustering of type species of genera in *Enterobacterales* based on pangenomic and phylogenomic analyses. Top, only the ribosomal genes were included in the phylogenomic analysis. Bottom, 71 single-copy genes were included in the phylogenomic analysis. Download FIG S5, EPS file, 0.2 MB. Copyright © 2020 Barco et al. 2020 Barco et al. This content is distributed under the terms of the Creative Commons Attribution 4.0 International license.

Interestingly, the type species of Morganella, *Moellerella*, Proteus, and *Providencia* are in borderline relationships to *Xenorhabdus* (Fig. 8), with their respective AF and ANI values in close proximity to the estimated inflection point. We note that pairwise 16S rRNA gene sequence identities between *X. nematophila* and Proteus vulgaris range from 94.5 to 95.0%, again a borderline case as far as genus assignment is concerned. It is noteworthy that the genus *Xenorhabdus* was proposed prior to the advent of 16S rRNA gene as a taxonomic marker. A more in-depth analysis of all these closely related genera and their potential taxonomic placement, especially in relation to *Proteus*, should be further investigated as more type strains in these taxa are sequenced.

**FIG 9:**
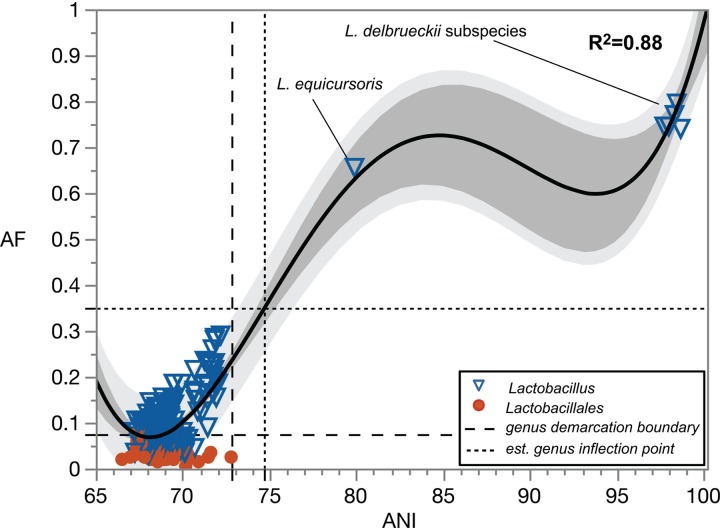
Pairwise genome comparisons to the type species of the genus *Lactobacillus*, *L. delbrueckii delbrueckii* DSM 20074^T^. Circles, type species (*n* = 38) of genera within the order *Lactobacillales*; triangles, non-type *Lactobacillus* spp. (*n* = 192). The regression line is based on quartic function, with the data set including the AF and ANI value of 1:100. The dark-gray-shaded area indicates the 95% confidence interval of the trendline. The light-gray-shaded area indicates the 95% confidence interval of AF and ANI values. The long dash line demarcates the genus boundary (left quadrant). The short dash line (left quadrant) extends from the estimated genus inflection point.

The last case we will analyze is the known polyphyletic *Lactobacillus*, a genus relevant to many subfields of microbiology, including food and public-health microbiology. *Lactobacillus sensu lato* has been proposed to have 24 phylogroups (47). More recently, *Lactobacillus* was proposed to contain 10 phylogroups (48). However, some difference in opinion to this interpretation has been documented (49), with a recommendation to apply more explicit criteria for the demarcation of a genus, in addition to the identification of phylogroup exclusive marker genes. Analysis of *Lactobacillus* following the approach herein presented, using *L. delbrueckii delbrueckii* as a primary reference, resulted in the identification of the genus demarcation boundary (AF, 0.075; ANI, 72.80%) and estimated genus inflection point (AF, 0.349; ANI, 74.71%; Fig. 9). Significant differences were seen between non-type species of *Lactobacillus* and type species of *Lactobacillales* in terms of AF (*P* < 0.0001) but not ANI (*P* = 0.1624), already highlighting profound taxonomic issues in this genus. This approach identified 73 *Lactobacillus* spp. that are below the genus demarcation boundary, which would strongly suggest reclassification of such species to other genera. A comparison to the GTDB taxonomy (Data Set S3C) shows almost (98%) complete agreement on the placement of these species into other genera (63 out of 64 species; 9 species could not be found on GTDB), with the exception of Lactobacillus floricola. However, the estimated genus inflection point is much higher than the current genus demarcation boundary, hinting at yet additional taxonomic discrepancies. Indeed, most of the *Lactobacillus* spp. (186 out of 193 species considered in this study) have AF and ANI values that fall below the estimated genus inflection point. Besides *L. delbrueckii* and its subspecies, only one other species is above the estimated genus inflection point, Lactobacillus equicursoris. Summarizing the results, *Lactobacillus sensu stricto* should currently only contain two species, *L. delbrueckii* and *L. equicursoris*. GTDB (22) and Salvetti et al. (48) currently have 40 (i.e., “d_Bacteria; p_Firmicutes; c_Bacilli; o_Lactobacillales; f_Lactobacillaceae; g_Lactobacillus”; GTDB accessed on 27 October 2019) and 33 (i.e., “*L. delbrueckii* group”) species within *Lactobacillus*, respectively. Some of the species in GTDB's “g_Lactobacillus” (22) and Salvetti et al.'s “*L. delbrueckii* group” (48) have very low AF values of <0.1 (e.g., *L. floricola* and L. iners), indicating <10% alignable region of best-bidirectional hits to the genome of *L. delbrueckii delbrueckii*, the type species of *Lactobacillus*. These results demonstrate that this genus is currently not being delineated taking into account a consistent aspect of genomic coherence, as more and more species are classified within *Lactobacillus* despite being as genetically distant from the primary reference as are other type species of genera in *Lactobacillale*s. Phylogenetically, *L. delbrueckii* and *L. equicursoris* form a monophyletic group, as seen by different methods in studies by Morita et al. (50), Zheng et al. (47), Sun et al. (51), Salvetti et al. (48), and Wittouck et al. (52). In terms of 16S rRNA identity, the only species that has an identity of >94.5% to *L. delbrueckii delbrueckii* is *L. equicursoris* (note that subspecies of *L. delbrueckii* have identities of >99%). All other *Lactobacillus* species in GTDB’s “g_Lactobacillus” (22) and Salvetti et al.’s “*L. delbrueckii* group” (48) have identities <94% to *L. delbrueckii delbrueckii*, with values as low as 86.9% (i.e., *L. floricola*; based on a comparison of 1,483 bp). These results highlight that there are still many taxonomic issues to resolve within *Lactobacillus*.

### Implications.

The approach we have used to demarcate genera is complementary and does not replace the conventional polyphasic method of circumscribing a genus, which includes thorough analyses of the full-length 16S rRNA gene, phylogeny, physiology, and metabolism, among other aspects. The AF and ANI boundary values for genus demarcation in a given family or order can be refined as the genomes of more type strains and type species are sequenced and become publicly available. This approach emphasizes the importance of type strains and type species in the continuous reevaluation of bacterial and archaeal taxa using genomic indices (e.g., ANI or AF) because these specimens function as reference points for their respective taxonomic groups and remain available in viable form from multiple public repositories, thus making the approach reproducible, replicable, robust, and generalizable (53). In this respect, our approach is in line with recommendations regarding taxonomy, including relevant comparisons to type strains and type species for the characterization of novel *Bacteria* and *Archaea* (8). It is reiterated that any rearrangement or reclassification of taxa should be in alignment with the ICNP (7). In particular, a potential rearrangement of type species must be carefully analyzed, as a genus can only contain one type species, and rules and principles of nomenclature must be followed in order to properly do that.

Uncultivated *Bacteria* and *Archaea* represented by metagenomic assembled genomes (MAGs) and/or single-cell amplified genomes (SAGs) could potentially be used in the implementation of this approach; however, it is noted that type strains and type species should be used as references for such analyses, if genomes are available (see “Guidelines,” below). The designation of type strains and type species is governed by the ICNP and requires a thorough characterization of the microorganism and the deposit of the type strain in at least two different culture collections in two different countries. Naturally, the proper characterization, and therefore, the type designation, is in most cases impossible for uncultivated *Bacteria* and *Archaea* given the current guidelines set by the ICNP. This could change if genomes are subsequently considered type material, as recently proposed (54) and discussed (55). In lieu of type strains and type species, proper and consistent genomic references must be established in order to analyze taxonomic groups without cultivated representatives as well as rigorous standards for sequence quality. Caution is advised in the interpretation of data resulting from low-quality MAGs since they can represent composite genomes of different strains (56). In theory, SAGs could serve as good taxonomic frames of reference for uncultivated genera; however, genome incompleteness in SAGs is an issue that would need to be addressed.

The MiSI method explicitly excludes tRNA and rRNA genes in order to avoid inflation of AF or ANI values. However, the current taxonomic framework is largely based on the use of the 16S rRNA gene for classification. Therefore, the approach herein investigated is not completely independent of the 16S rRNA gene. The fact that in the majority of the groups tested, with the exception of known polyphyletic genera such as *Bacillus* (although significant differences between type and non-type species were still detected in these groups), there was strong concordance of the type species of genera being delineated from non-type species of the primary reference provides evidence to the success of the use of the 16S rRNA gene within the taxonomic framework, as far as precision is concerned (i.e., not accuracy). The 16S rRNA gene has served and could continue to serve the scientific community well, especially in maintaining relative consistency in classifications. However, the estimation of the genus inflection point indicates that the 16S rRNA gene identity minimum threshold value of 94.5% could underestimate genera diversity in some taxa (e.g., *Bacillaceae* [*Bacillus*]) and overestimate it in others (e.g., *Enterobacterales* [*Photorhabdus*]), especially if multiple nonidentical 16S rRNA genes are present in the genomes. This uncertainty in delineating genera using the 16S rRNA gene has also been shown by Yarza et al. (3), considering that the minimum and median identity values for delineation differ by nearly 2%, at 94.5% and 96.4%, respectively. It is clear that different taxonomic groups are characterized by different AF and ANI threshold values for genus demarcation. However, even within established taxonomic groups, the effective AF and ANI threshold values will still be “moving targets,” dependent on the assignment and reassignment of new type species. Thus, a single, universal, stationary threshold for genus delineation will not be able to sensitively resolve genus assignments for all taxa.

The reader is made aware that variations in bidirectional, matched-pair AF values (AF1-2 and AF2-1) exist. These variations are usually small but could be relevant in the interpretation of data, especially in borderline cases. Bidirectional, matched-pair ANI values (ANI1-2 and ANI2-1) differ by <0.3% in the majority of the cases, which would make the error bars invisible to the naked eye. A representative figure with corresponding standard deviation values for AF is included in the supplemental material (Fig. S6).

10.1128/mBio.02475-19.10 FIG S6 Pairwise genome comparisons to the type species of the genus *Flavobacterium*, *F. aquatile* LMG 4008^T^ (Fig. 4b). Circles, type species (*n* = 71) of genera within the family *Flavobacteriaceae*; triangles, non-type species (*n* = 82) of the genus *Flavobacterium*. The bottom-left quadrant demarcates the boundary between type and non-type species. Error bars indicate one standard deviation from the mean. Download FIG S6, EPS file, 1.3 MB. Copyright © 2020 Barco et al. 2020 Barco et al. This content is distributed under the terms of the Creative Commons Attribution 4.0 International license.

The use of ANI has been previously stated as not applicable for the demarcation of genera (14, 18, 19, 57). Our results indicate that the MiSI method, and therefore AF and ANI, can be used to visualize natural breakpoints that can be used to circumscribe genera with objectivity, reproducibility, and high resolution if the guidelines presented in this study are followed. Our study identified potentially misclassified species in numerous genera that could not be previously resolved by alternative methods, including DNA-DNA hybridization, 16S rRNA gene-based phylogeny, and phylogeny using concatenated highly conserved proteins. Adaptation of this method to demarcate higher taxonomic ranks has not been tested, as it is beyond the scope of this study. Nonetheless, such an approach would be warranted. Finally, we highlight that the results demonstrate a conserved genomic coherence at the genus level for numerous different taxa, shedding light on a fundamental emergent property of *Bacteria* and *Archaea*.

## MATERIALS AND METHODS

### ANI and AF.

ANI and alignment fraction (AF) values were obtained by the Microbial Species Identifier (MiSI) method using ANIcalculator 2014-127, version 1.0 (https://ani.jgi.doe.gov/html/home.php?page=introduction) (17), and also as temporarily implemented in the JGI-Integrated Microbial Genomes and Microbiomes (IMG/M) system (https://img.jgi.doe.gov/) via the Pairwise ANI tool (accessed 2017 to 2018). ANI, as defined by Varghese et al. (17), is calculated for a pair of genomes by averaging the nucleotide identity of orthologous genes identified as bidirectional best hits (BBHs), which are the genes that show ≥70% sequence identity and ≥70% alignment of the shorter gene. AF, as defined by Varghese et al. (17), is calculated as a fraction of the sum of the lengths of BBH genes divided by the sum of the lengths of all genes in a genome.

### Strains.

Unless otherwise noted, only type strains were used in this study. The NamesforLife Database (NamesforLife, LLC, East Lansing, MI [58]) was primarily used to retrieve nomenclature (current up to 27 October 2019), nomenclatural history, and taxonomic information about validly published type strains and type species associated with different genera. Complementary to this, equivalent strain numbers assigned by different biological resource centers (e.g., the German Collection of Microorganism and Cell Cultures [DSMZ]) were searched in their respective online catalogues. In addition to what is mentioned above, primary taxonomic literature sources were used to confirm some of these designations. All strain designations were cross-referenced in at least two databases.

### Genomes and 16S rRNA gene sequences.

Publicly available genomes were obtained from IMG and the National Center for Biotechnology Information (NCBI; https://www.ncbi.nlm.nih.gov/). Generally, taxonomic orders/families that have genomes of ≥4 type species of genera and ≥4 non-type species in a given genus were considered. Genomes that were unanimously flagged by both IMG and the NCBI as low quality were removed from the data set. About 36% of the genomes included in this study were sequenced as part of the JGI-Genomic Encyclopedia of *Bacteria* and *Archaea*, a project that focuses on sequencing the genomes of type strains (5, 59). Sequences of the 16S rRNA gene were obtained from either IMG, the NCBI, or EZBioCloud (60; https://www.ezbiocloud.net/). Alignments were separately performed using the SILVA incremental aligner version 1.2.11 (61; https://www.arb-silva.de/aligner/) and ClustalW via the Geneious platform (version R6; Biomatters, Auckland, New Zealand). Genetic distances were calculated in Geneious. PhyML (62) was used via the Geneious platform to generate the maximum likelihood phylogenetic tree with the following settings: Hasegawa-Kishino-Yano (HKY85) substitution model, 1,000 bootstraps, estimated transition/transversion ratio, estimated proportion of invariable sites, estimated gamma distribution, and branch lengths and substitution rate optimized.

### Pangenome analysis.

Pangenome analysis of *Bacillus* and other genera in *Bacillaceae* were processed in anvi’o (version 5.5) (63) following the workflow for microbial pangenomics (http://merenlab.org/2016/11/08/pangenomics-v2/, last accessed 13 May 2019 [64]). In brief, we generated contig databases for each genome contig file using the command “anvi-gen-contigs-database.” Prodigal (65) was used to identify open reading frames, and subsequently, each database was populated with HMM profiles by comparison to a collection of single-copy genes using HMMER (66). Once contig databases were generated for all genomes, we used “anvi-gen-genomes-storage” to generate a master genome storage database to use in the pangenome analysis. We used the NCBI-BLAST option in “anvi-pan-genome” to calculate gene similarity and MCL (67) for clustering under the following settings: minbit, 0.5; mcl inflation, 2; and minimum occurrence, 2. For phylogenomic analysis of *Bacillus* genomes, we selected a subset of 21 genes based on the following criteria: minimum number of genomes in which gene cluster occurs, 35; maximum number of genes from each genome, 1; maximum functional homogeneity index, 0.9; and minimum geometric homogeneity index, 0.99. We then used the anvi’o command “anvi-get-sequences-for-gene-clusters” to concatenate and align target genes from all genomes and “anvi-gen-phylogenomic-tree” to generate the phylogenomic tree from the concatenated FASTA file. The tree was then rerooted using other genera of *Bacillaceae* as the outgroup.

### Statistical analysis.

All bidirectional, matched-pair values of ANI (ANI1-2 and ANI2-1) and AF (AF1-2 and AF2-1) were reported as single averaged ANI and AF values in this study. The nonparametric Wilcoxon test was performed separately for each set of ANI and AF values to determine significant (*P* < 0.05) differences between type and non-type species. All statistical analyses were performed using the statistical software JMP Pro, version 14 (SAS Institute, Inc.).

### Rationale.

The nomenclatural type or “type” is defined by the International Code of Nomenclature of Prokaryotes (ICNP) as “that element of the taxon with which the name is permanently associated …” (rule 15 [7]). The type strain acts as the single reference for a given species. Each species and subspecies with a validly published name has a type strain that is designated at the time the name is proposed. The vast majority (>98%) are represented by one or more viable deposits that are descended from the original type strain, are maintained in pure culture, agree closely with its character in the original description, and are available in one or more public culture collections (rules 18a and 30 [7]). A type species, which is represented by its type strain, acts as the single reference for a given genus. Type strains and type species may not necessarily be the most representative members of a species or genus, respectively. Rather, type strains and type species represent the first and often only member of the respective taxa, based on the opinion of the individual proposing each name. The rationale used in this study is that sister species in a given genus should be relatively similar to the type species of the cognate genus, indicating high similarity at the genome level. Therefore, when a type species of a genus is compared to a non-type species within the same genus, the AF and ANI values should be relatively high. In contrast to this, type species of different sister genera (i.e., in the same taxonomic family or order) should be relatively dissimilar at the genome level. This dissimilarity should be reflected in relatively low AF and ANI values.

### Guidelines.

In addition to the genome(s) under consideration (e.g., of a cultured strain that needs classification), at least two genomes of type species in the same family or order are needed, as follows: (i) the primary reference which is the phylogenetically closest relative that is a type species (i.e., the type species to which all other microbes will be compared), and (ii) all other type species (≥1) in the same family or order. Furthermore, all available genomes of non-type species that belong to the same genus of the primary reference are needed. In order for the results to be meaningful, a species must only be represented by the type strain unless its genome is not available, in which case a non-type strain of the same species that is ≥99% identical in 16S rRNA gene sequence(s) (i.e., all copies of 16S rRNA gene in the genome) may be used as a proxy until a genome becomes available. The accession numbers for these genomes should be reported. The genus demarcation boundary is set by the highest AF and ANI values of a type species (i.e., other than the primary reference) in an order/family.

### Interpretation.

Every data point in the AF and ANI plot is calculated in relation to the primary reference, which is defined above as the type strain of the type species of the specific genus to be analyzed; therefore, the plot represents genomic similarity to the reference genome. If the primary reference is compared against an identical genome (e.g., or a direct comparison against itself), an AF value of 1 and ANI value of 100 would result. If the primary reference is compared against relatively dissimilar genomes, the AF and ANI values will reflect proportionally lower values. Thus, the plots that are generated effectively illustrate how distant other data points are from the primary reference genome in terms of AF and ANI values, or in other words, how distant other data points are from the upper-right corner (AF, 1; ANI, 100%).

When all comparisons are made in relation to the primary reference, a type species (i.e., other than the primary reference) should always cluster with other type species of genera in the same taxonomic family or order, reflecting relatively low AF and ANI values. A non-type species should always cluster with other non-type species of the same genus, reflecting relatively high AF and ANI values. The distinct clustering of type species and non-type species should be conserved without any overlap. If a non-type species is positioned within the cluster of type species in the same taxonomic family or order (i.e., at or below the genus demarcation boundary), this indicates that the non-type species could potentially be assigned to a different genus, as it is as genomically different from the primary reference, as are other type species in the same taxonomic family or order. If a type species is positioned within the cluster of non-type species, there is support for potential reclassification of the type species within the same genus of the primary reference, given the accumulation of other supporting evidence (e.g., 16S rRNA gene identity or phylogeny). It is emphasized that any proposed taxonomic rearrangement or reclassification based on AF and ANI results will necessarily affect nomenclature; therefore, any corresponding changes in nomenclature resulting from such proposal must be in alignment with the ICNP (7).

10.1128/mBio.02475-19.1 TEXT S1 Pangenome analysis of *Xenorhabdus* and *Photorhabdus*. Download Text S1, DOCX file, 0.1 MB. Copyright © 2020 Barco et al. 2020 Barco et al. This content is distributed under the terms of the Creative Commons Attribution 4.0 International license.
