# Supplementary material for: A Genus Definition for Bacteria and Archaea Based on a Standard Genome Relatedness Index
Source: mBio. 2020 Jan 14;11(1):e02475-19. doi: 10.1128/mBio.02475-19 (PMC6960282; doi:10.1128/mBio.02475-19)
Supplement: TEXT S1 [file mBio.02475-19-s0001.docx]

Supplemental Section: A genus definition for *Bacteria* and *Archaea* based on a standard genome relatedness index.

Barco, R.A.^1,2,5*^, Garrity, G.M.^3^, Scott, J.J.^4^, Amend, J.^1,2^, Nealson, K.H.^1,2^, and Emerson, D.^5^

^1^Department of Earth Sciences, University of Southern California, Los Angeles, CA, USA.

^2^Department of Biological Sciences, University of Southern California, Los Angeles, CA, USA.

^3^Department of Microbiology and Molecular Genetics, Michigan State University, East Lansing, MI, USA.

^4^Smithsonian Tropical Research Institute, Panama, Republic of Panama.

^5^Bigelow Laboratory for Ocean Sciences, East Boothbay, ME, USA.

*Pangenome Analysis of Xenorhabdus and Photorhabdus*

Pangenome analysis of *Xenorhabdus*, *Photorhabdus* and other genera in *Enterobacterales* were processed in anvi'o (version 5.5) following the workflow for microbial pangenomics ([merenlab.org/2016/11/08/pangenomics-v2/](https://urldefense.proofpoint.com/v2/url?u=http-3A__merenlab.org_2016_11_08_pangenomics-2Dv2_&d=DwMFaQ&c=clK7kQUTWtAVEOVIgvi0NU5BOUHhpN0H8p7CSfnc_gI&r=bMr4RGqtSowoCMT-VMxOWA&m=hvRSPxulPIzatu8wsOngPf_GDEBDSNuTG6BC6-p_IWc&s=gCbN0NMg20fN13Nyk-vNW3QzLzVwHX6IO_m0SMuSjk4&e=)). In brief, we generated contig databases for each genome contig file using the command 'anvi-gen-contigs-database'. Prodigal was used to identify open reading frames and subsequently each database was populated with HMM profiles by comparison to a collection of single-copy genes using HMMER. Once contig databases were generated for all genomes, we used 'anvi-gen-genomes-storage' to generate a master genome storage database to use in the pangenome analysis. We used the ncbi-blast option in 'anvi-pan-genome' to calculate gene similarity and MCL for clustering under the following settings: minbit, 0.5; mcl inflation, 2; minimum occurrence, 2. For phylogenomic analysis of *Xenorhabdus and Photorhabdus* genomes, we used both the full collection of 71 single-copy genes and the subset of 36 ribosomal genes. We used the anvi’o command ‘anvi-get-sequences-for-gene-clusters’ to concatenate and align target genes and ‘anvi-gen-phylogenomic-tree’ to generate the phylogenomic trees from the concatenated FASTA files.

Dataset S1. List of genomes used in this study. Taxa in bold indicate the taxonomic resolution at which the group was analyzed.

Dataset S2. a) Summary of taxonomic groups and genomes included in this study. Taxa in bold indicate the taxonomic resolution at which the group was analyzed. For a detailed species list including accession numbers, see Dataset S1. b) AF and ANI mean and median values associated with genus demarcation boundaries in *Archaea* and *Bacteria*. c) List of estimated genus inflection points. For accuracy, this list only includes the genus inflection points that were consistent with Gompertz and Logistic regressions and had, at a minimum, R^2^ values approximating 0.90 (quartic regression).

Dataset S3. Comparisons of species designations to GTDB taxonomy. a) Species composition of *Bacillus* based on MiSI and GTDB (only considering type strains). b) Species composition of *Clostridium* based on MiSI and GTDB (only considering type strains, unless noted otherwise). c) Species composition of *Lactobacillus* based on MiSI and GTDB (only considering type strains with available genomes).

Figure S1. Distribution of AF and ANI values of type (T; n=82) and non-type (NT; n=96) species when compared to a primary reference in their respective order/family in *Archaea*. The green line indicates the mean. The blue lines show the standard deviation from the mean.

Figure S2. AF (a and b) and ANI (c and d) values of type (T; n=2382) and non-type species (NT; n= 2571) when compared to primary references within specific taxonomic orders in *Bacteria*.

Figure S3. Distribution of AF and ANI values in *Archaea* at the order (a-d) and family (e-h) levels. AF (a and b) and ANI (c and d) values of non-type species (n=96) and type species (n=82) when compared to primary references within specific taxonomic orders in *Archaea*. AF (e and f) and ANI (g and h) values of type (T; n=69) and non-type species (NT; n= 73) when compared to primary references within specific taxonomic families in *Archaea*.

Figure S4. Pairwise genome comparisons of *Thiomicrospira* and *Thiomicrorhabdus*. a) Pairwise genome comparisons to the type species *Thiomicrospira pelophia* DSM 1534^T^ using the old classification scheme (*i.e*., prior to rearrangement by Boden et al. [35]) show clear taxonomic issues and no distinct clustering between type and non-type species. Non-type species within the genus *Thiomicrospira* are shown in triangles. Type species of genera within the family *Piscirickettsiaceae* are shown in circles. Inset: zoomed-in boxed area. b) Pairwise genome comparisons to *Thiomicrorhabdus* sp. KP2 (> 99% pairwise identity to the 16S rRNA gene sequence of the type species *Tmr. frisia* JB-A2^T^, which does not have a sequenced genome) using the new re-classification scheme by Boden *et al.* (35). Non-type species within the genus *Thiomicrorhabdus* are shown in triangles. Type species of genera within the family *Piscirickettsiaceae* are shown in circles. The bottom-left quadrant demarcates the boundary between type and non-type species. c) Pairwise genome comparisons to *Thiomicrospira pelophila* DSM 1534^T^ using the new re-classification scheme by Boden *et al*. (35). Non-type species within the genus *Thiomicrospira* are shown in triangles. Type species of genera within the family *Piscirickettsiaceae* are shown in circles, with the exception of *Thiomicrorhabdus frisia* JB-A2^T^ which does not have a sequenced genome; *Thiomicrorhabdus* sp. KP2 (> 99% pairwise identity to the 16S rRNA gene sequence) was used instead. The bottom-left quadrant demarcates the boundaries between type and non-type species.

Figure S5. Genome clustering of type species of genera in *Enterobacterales* based on pangenomic and phylogenomic analysis. Top: only the ribosomal genes were included in the phylogenomic analysis. Bottom: 71 single-copy genes were included in the phylogenomic analysis.

Figure S6. Pairwise genome comparisons to the type species of the genus *Flavobacterium*: *F. aquatile* LMG 4008^T^ (Figure 4b). In circles: type species (n=71) of genera within the family *Flavobacteriaceae*. In triangles: non-type species (n=82) of the genus *Flavobacterium*. The bottom-left quadrant demarcates the boundary between type and non-type species. Error bars indicate one standard deviation from the mean.
